# Lurbinectedin in extensive-stage small-cell lung cancer: a brief report of the IFCT-2105 LURBICLIN study☆

**DOI:** 10.1016/j.esmoop.2024.103968

**Published:** 2024-11-27

**Authors:** N. Girard, F. Guisier, A. Swalduz, S. Van Hulst, E. Pichon, P. Lavaud, L. Greillier, A. Tiotiu, A. Madroszyk, O. Bylicki, A. Canellas, L. Belmont, M. Zysman, P.-A. Hauss, B. Godbert, C. Audigier-Valette, C. Lebreton, F. Morin, V. Westeel

**Affiliations:** 1 Department of Medical Oncology, Institut Curie, Paris, France; 2 Paris Saclay University, UVSQ, Versailles, France; 3 Normandie Univ, UNIROUEN, LITIS Lab QuantIF team EA4108, CHU Rouen, Rouen, France; 4 Inserm CIC-CRB 1404, Rouen, France; 5 Centre Léon Bérard, Lyon, France; 6 CHU Nîmes, Nîmes, France; 7 CHRU Bretonneau, Tours, France; 8 Gustave Roussy, Paris-Saclay University, Villejuif, France; 9 APHM, Hôpital Nord, AMU, Marseille, France; 10 CHU de Brabois, Vandoeuvre-Les-Nancy, France; 11 Institut Paoli-Calmettes, Marseille, France; 12 HIA Sainte Anne, Toulon, France; 13 École du Val de Grâce, Paris, France; 14 APHP Hôpital Tenon, Paris, France; 15 Centre Hospitalier Victor Dupouy, Argenteuil, France; 16 CHU, Hôpital Haut-Lévèque, Pessac, France; 17 Centre Hospitalier Intercommunal Elbeuf Louviers, Elbeuf, France; 18 Hôpital Robert Schuman, UNEOS, Metz, France; 19 CHITS Toulon Sainte Musse, Toulon, France; 20 The French Cooperative Thoracic Intergroup, Paris, France; 21 CHU Besançon, Hôpital Minjoz, Besançon, France

**Keywords:** lurbinectedin, small-cell lung cancer, chemotherapy, immunotherapy, compassionate use trials

## Abstract

**Background:**

Small-cell lung cancer (SCLC) is a highly aggressive type of lung cancer. Lurbinectedin is recommended as second-/third-line treatment for advanced, previously treated SCLC.

**Materials and methods:**

LURBICLIN is a nationwide, non-interventional, retrospective chart review study, based on the cohort of consecutive patients enrolled in the named patient use for lurbinectedin in France.

**Results:**

A total of 312 patients were included. Lurbinectedin was delivered as second-line therapy in 138 (44%) patients. Grade 3-4 treatment-related adverse events were observed in 28 (9%) and 15 (5%) patients, respectively. Objective response rate (ORR) to lurbinectedin was 22% in the intention-to-treat population. After a median follow-up of 20.8 months, median progression-free survival (PFS) was 1.9 months [95% confidence interval (CI) 1.8-2.0 months]. At multivariate analysis, chemotherapy-free interval (CTFI) ≥ 90 days was an independent predictor of higher PFS [hazard ratio (HR) = 0.64, 95% CI 0.50-0.84, *P* < 0.0001]. The median overall survival (OS) was 4.7 months (95% CI 4.0-5.4 months). At multivariate analysis, performance status < 2 and CTFI ≥ 90 days were independent predictors of higher OS (HR = 0.71, 95% CI 0.53-0.95, *P* = 0.03; and HR = 0.58, 95% CI 0.44-0.76, *P* < 0.0001, respectively). Overall, 147 (47%) patients had initiated subsequent systemic treatments.

**Conclusions:**

LURBICLIN confirms the activity of lurbinectedin in patients with SCLC with a manageable safety profile. Lurbinectedin monotherapy provides an alternative option for SCLC patients.

## Highlights

• Lurbinectedin is recommended as second-/third-line treatment for advanced, previously treated SCLC.
• LURBICLIN is a nationwide, non-interventional cohort of consecutive patients enrolled who received lurbinectedin.
• Lurbinectedin was mostly delivered in a late-line setting, with manageable safety profile with full dosing.
• Efficacy, especially ORR and OS, were numerically lower with that reported in the landmark basket phase II trial.
• Concurrent or subsequent radiotherapy was frequently delivered, and many patients were eligible to subsequent therapies.

## Introduction

Small-cell lung cancer (SCLC) is a highly aggressive type of lung cancer with rapid tumor growth and progression in a majority of patients after first-line treatment for extensive-stage disease.^1^ The standard of care in such a situation is rechallenge of platinum-etoposide chemotherapy in so-called ‘platinum-sensitive’ cases with chemotherapy-free interval (CTFI) ≥ 90^2^ or 180 days,^3^ topotecan, or CAV (cyclophosphamide, doxorubicin, vincristine), among other single-agent regimes.^2^^,^^3^ Overall, novel options are needed for patients after the failure of standard first-line chemotherapy and immunotherapy combination.

Lurbinectedin is recommended after progression to first-line chemotherapy for advanced, metastatic SCLC based on the results of a landmark basket single-arm, phase II trial conducted in 105 patients, which reported an objective response rate (ORR) by investigator assessment of 35% [95% confidence (CI) 26% to 45%].^4^ This led lurbinectedin to be granted accelerated approval by the Food and Drug Administration as well as being included in international guidelines as one of the preferred options in this setting.^2^^,^^3^ Meanwhile, the phase III, randomized ATLANTIS trial failed to demonstrate overall survival (OS) improvement with a combination of lurbinectedin plus doxorubicin versus control in this setting.^5^ Lurbinectedin was granted an approval in several countries, while in other countries access to lurbinectedin is possible mainly under named patient use (NPU).

While additional prospective trials are still ongoing, there is a need to better assess the efficacy of lurbinectedin in large, well-defined cohorts of patients with SCLC.

## Materials and methods

### Study design and inclusion criteria

In the nationwide, non-interventional, retrospective chart review InterGroupe Francophone de Cancérologie Thoracique (IFCT)-2105 LURBICLIN study, we analyzed a large, multicenter, cohort of consecutive patients enrolled in the NPU (*Autorisation Temporaire D’Utilisation Nominative* – *ATU Nominative*) according to regulatory French terminology by the time of the data collection for lurbinectedin in France from June 2020 to March 2021; all patients who received at least one dose of treatment and gave their consent for the data collection were enrolled from 47 sites. Based on onsite visits, the data collection period ran from July 2022 to December 2022, by trained IFCT clinical research associates. This research was registered in the Health Data Hub public directory (https://www.health-data-hub.fr/projets) and in clinicaltrials.gov database under the ID NCT05285033.

### Lurbinectedin treatment

As per the French NPU, patients had to receive lurbinectedin at the dose of 3.2 mg/m^2^ administered as a 1-h intravenous infusion every 3 weeks until disease progression or unacceptable toxicity.

### Study endpoints

The primary endpoint was to describe the clinical characteristics of patients, and secondary endpoints included exposure to treatment, best response, real-world progression-free survival (PFS), OS, patterns of tumor progression, treatment sequences, and safety. Key pre-specified subgroups included treatment line and CTFI ≥ 90 days versus <90 days.^2^^,^^3^ Last follow-up was on 1 September 2022.

## Results

### Patient population

A total of 312 patients were included. Patient characteristics are presented in Table 1. Briefly, majority of the patients were men (*n* = 200, 64%) and those with a performance status (PS) of 0 or 1 (*n* = 188, 72%). Lurbinectedin was delivered as second-line therapy in 138 (44%) patients, and as later line in 174 (56%) patients. CTFI was <90 days—the so-called resistant cases—in 164 (58%) patients, and <30 days—the so-called refractory cases—in 45 (16%) patients. Metastatic sites included the lung in 277 (89%) patients, the mediastinum in 215 (69%), the liver in 149 (48%) patients, the brain in 147 (47%) patients, and the bone in 115 (37%) patients. A majority of patients (*n* = 180, 58%) had previously received an immunotherapy-based regimen.

**Table 1:** Patients’ characteristics

		*N* (%)
Total		312 (100)
Median age		65.4 years
Gender	Male	200 (64)
	Female	112 (36)
Smoking	Yes	298 (96)
	No	14 (5)
Initial stage at diagnosis	Extensive	268 (86)
	Limited	44 (14)
Performance status at lurbinectedin initiation	0-1	188 (72)
	≥2	74 (28)
	Unknown	50
Brain metastasis at lurbinectedin initiation	Yes	147 (47)
	No	165 (53)
Previous lines of systemic therapy	1	138 (44)
	2	93 (30)
	3	50 (16)
	>3	31 (10)
Received at least one immunotherapy during previous line(s)		180 (58)
Received at least one chemotherapy during previous line(s)		283 (91)
Treatment sequence platinum–platinum rechallenge–lurbinectedin		45 (14)
Treatment sequence platinum–lurbinectedin–platinum rechallenge		20 (6)
Free interval since the last antineoplastic treatment received (mean ± SD, months)		2.4 ± 3.1
CTFI < 90 days (ESMO guideline)		164 (58)
CTFI ≥ 90 days (ESMO guideline)		119 (42)
CTFI <180 days (NCCN guidelines)		256 (91)
CTFI ≥180 days (NCCN guidelines)		27 (10)

CTFI, chemotherapy-free interval; ESMO, European Society for Medical Oncology; NCCN, National Comprehensive Cancer Center; SD, standard deviation.

### Lurbinectedin treatment

A median number of 3 cycles (range 1-24) of lurbinectedin were administered. Grade 3-4 treatment-related adverse events (TRAEs) were observed in 28 (9%) and 15 (5%) patients, respectively (Supplementary Table S1, available at https://doi.org/10.1016/j.esmoop.2024.103968). A total of 38 dose reductions from the full 3.2 mg/m^2^ dosing were observed along with treatment delivery in 20 (6%) patients, mostly (76% of cases) related to TRAEs or intercurrent events (24% of cases). Dose delays were observed in eight (3%) patients in the context of TRAEs (three cases), delivery of radiotherapy (two cases), and intercurrent event (three cases). Concurrent radiotherapy was delivered in 70 (22%) patients, including 48 (15%) who received brain irradiation, and 22 (7%) who received palliative-intent radiotherapy on other sites including the bone, the lymph nodes, the primary tumor, or the adrenal.

At the cut-off date, 311 had discontinued lurbinectedin because of disease progression (*n* = 259, 83%), death (*n* = 24, 8%), toxicity (*n* = 15, 5%), investigator decision (*n* = 10, 3%), or other reasons (*n* = 3, 1%).

### Efficacy outcomes

**Figure 1:**
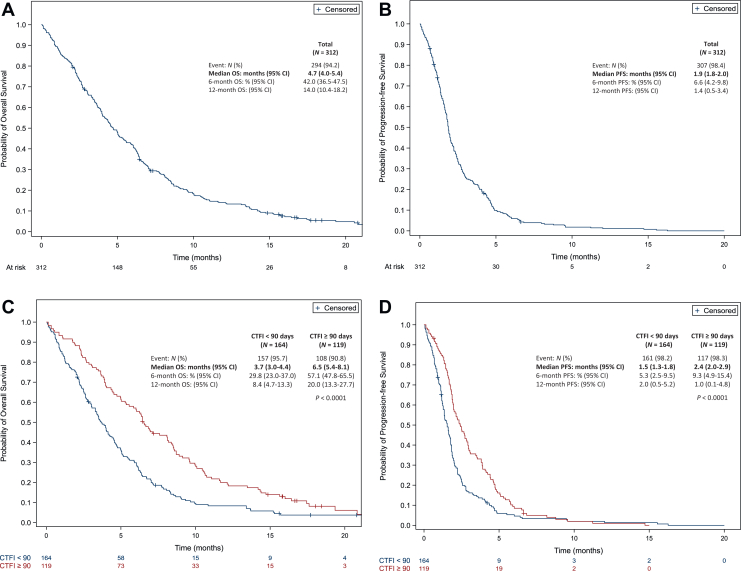
(A) Overall survival and (B) progression-free survival in 312 patients who received lurbinectedin. (C) Overall survival and (D) progression-free survival according to chemotherapy-free interval. Red is CTFI > 90 days. CI, confidence interval; CTFI, chemotherapy-free interval; OS, overall survival; PFS, progression-free survival.

The ORR to lurbinectedin was 22%, and disease control rate (DCR) was 38% (Table 2); the CTFI ≥ 90 days group was associated with numerically higher ORR and DCR of 27.3% and 44.5%, respectively. After a median follow-up of 20.8 months, the median PFS was 1.9 months (95% CI 1.8-2.0 months) (Figure 1B). At multivariate analysis, CTFI ≥ 90 days was an independent predictor of higher PFS (and HR = 0.64, 95% CI 0.50-0.84, *P* < 0.0001) (Figure 1D).

**Table 2:** Best response according to investigators

	All patients	CTFI <90 days	CTFI ≥90 days
	(*N* = 312)	(*N* = 164)	(*N* = 119)
Complete response	1 (0.4%) (0%-1.1%)	0	1 (1%) (0%-3%)
Partial response	60 (22%) (17%-27%)	23 (17%) (10%-23%)	29 (26%) (18%-35%)
Objective response	61 (22%) (17%-27%)	23 (17%) (10%-23%)	30 (27%) (19.0%-36%)
Stable disease	43 (16%) (11%-20%)	18 (13%) (7%-19%)	19 (17.3%) (10.2%-24.3%)
Disease control	104 (38%) (32%-44%)	41 (30%) (22%-37%)	49 (44.5%) (35.3%-53.8%)
Progression disease	168 (61%) (56%-67%)	97 (70%) (63%-78%)	59 (53.6%) (44.3%-63.0%)
Not evaluable	2 (1%) (0%-2%)	0	2 (1.8%) (0%-4.3%)
Not done/missing	38	26	9

CTFI, chemotherapy-free interval.

The median OS was 4.7 months (95% CI 4.0-5.4 months) (Figure 1A). At multivariate analysis, PS < 2 and CTFI ≥ 90 days were independent predictors of higher OS (HR = 0.71, 95% CI 0.53-0.95, *P* = 0.03; and HR = 0.58, 95% CI 0.44-0.76, *P* < 0.0001, respectively) (Figure 1C).

There were no significant differences in lurbinectedin-related outcomes based on age, number or regimen of prior lines of treatment, or presence of brain metastases. Of note, intracranial PFS was 3.1 months (95% CI 2.6-4.1 months) and 8.8 months (95% CI 4.9 months-not reached) in patients with and without baseline brain metastases, respectively.

### Post-lurbinectedin treatments

The most frequent sites of disease progression after lurbinectedin were lung (*n* = 115, 39%), brain (*n* = 114, 39%), liver (*n* = 88, 30%), mediastinum (*n* = 87, 30%), and bone (*n* = 45, 15%). At last follow-up, a total of 147 (47%) patients had initiated subsequent systemic therapy after discontinuation of lurbinectedin, which consisted of topotecan (*n* = 38, 26%), platinum-based chemotherapy (*n* = 34, 23%), adriamycin-based chemotherapy (*n* = 33, 23%), single-agent chemotherapy (*n* = 39, 27%), or immune checkpoint inhibitor (*n* = 3, 2%). Subsequent radiotherapy was delivered to 34 (22%) of these patients. ORR, DCR, and median PFS with first subsequent treatment after lurbinectedin were 11%, 35%, and 1.9 months (95% CI 1.7-2.3 months), respectively.

## Discussion

IFCT-2105 LURBICLIN is the largest study with single-agent lurbinectedin in extensive stage-SCLC and provides new insights for its actual use within the treatment algorithm, together with key efficacy outcomes as follows: (i) lurbinectedin was mostly delivered in a late-line setting for platinum-resistant patients, with manageable safety profile with full dosing; (ii) efficacy outcomes, especially ORR and OS, are numerically lower with that reported in the landmark basket phase II trial which included only second-line patients without brain metastases; and (iii) there was a relatively high use of concurrent or subsequent radiotherapy despite the burden of the disease, as well as a significant chance of subsequent therapy despite the late-line setting.

Unlike the phase II study, in our cohort, lurbinectedin was only primarily delivered in a second-line setting in 44% of patients, and there were 58% of platinum-resistant patients. This fits with ESMO clinical practice guidelines for SCLC, which recommend lurbinectedin for these patients, including refractory and PS 2 patients, which accounted for 16% and 28% of patients in our cohort, respectively; in addition, 47% of patients had brain metastasis.^2^ In the landmark phase II trial,^4^ there were 93% of patients treated in a second-line setting, including only 8% PS 2 patients, 21% of patients with refractory disease, and 4% of patients with brain metastases.

Given these differences in well-known prognostic factors in SCLC,^6^ some key efficacy outcomes in LURBICLIN were lower than that reported in the landmark basket trial: ORR of 17% versus 22.2% in platinum-resistant patients, 27% versus 45% in platinum-sensitive patients, PFS of 1.5 versus 2.6 months, and 2.4 versus 4.6 months, respectively. OS was also lower in LURBICLIN: 3.7 months in platinum-resistant, and 6.5 months in platinum-sensitive, when this was 5.0 and 11.9 months, respectively, in the trial. Still, the figures reported in LURBICLIN are deemed to be higher than that of other available agents used in that setting, such as topotecan, based on recent trials.^5^^,^^7^ Ultimately, our results are in line with that reported from smaller real-word cohorts of patients.^8^ Of note, the subgroup analysis of the landmark basket phase II trial with lurbinectedin that excluded chemotherapy-refractory patients reported a 41% ORR, with a 5.3-month duration of response, and a 10.2-month OS.^9^

As safety outcomes in LURBICLIN were similar to that reported in the trial—with a treatment-related discontinuation rate as low as 2%, the higher burden of disease in later lines of treatment in SCLC may also have had a major impact on the assessment of these endpoints. Ultimately, the ongoing LAGOON trial is aiming at randomizing 705 patients with relapsed SCLC to receive lurbinectedin (alone or in combination with irinotecan) versus topotecan or irinotecan,^10^ with stratification on CTFI and brain metastases, with the exclusion of patients with refractory disease. As in LURBICLIN, it is expected that most patients will be previously exposed to immunotherapy-based chemotherapy regimens. Interestingly, biomarkers may help to select SCLC patients with a higher chance of efficacy of lurbinectedin: preclinical studies showed that a low SLFN11 expression is predicting relative resistance to lurbinectedin, with potential induction of synthetic lethality with ATR inhibitors.^11^

Another key finding in LURBICLIN that may be of interest for clinical practice is the frequent use of concurrent or subsequent radiotherapy—delivered to 22% of patients in our cohort, mostly to the brain, despite the high burden of the disease. This was previously reported during first-line chemotherapy with or without immunotherapy, but mostly in a setting of oligoprogressive disease allowing continuation of treatment.^12^^,^^13^ Here, radiotherapy was mostly delivered concurrently, possibly to improve metastasis-related symptoms. Still, radiotherapy was not associated with a higher efficacy or toxicity in our cohort. Interestingly, after lurbinectedin, both multisite and oligoprogression may be observed; given the retrospective nature of the study, this was not formally collected in LURBICLIN. Radiotherapy was delivered to 22% of patients as subsequent therapy, suggesting a pattern of oligoprogressive disease.

Ultimately, our results show that, among the 49% of patients who initiated subsequent systemic therapy (with a preferred use of topotecan and platinum-based regimens), PFS outcomes were quite similar to those reported with lurbinectedin. This highlights the feasibility of subsequent chemotherapy, but the need for additional options in the late-line setting for SCLC patients.

To conclude, our real-world data confirmed the activity of lurbinectedin in patients with SCLC with a manageable safety profile. Activity remains modest in patients with PS2, brain metastases, and a CTFI < 90 days. Lurbinectedin monotherapy provides an alternative therapeutic option for SCLC patients without precluding subsequent therapies.
