# Supplementary material for: Lurbinectedin in extensive-stage small-cell lung cancer: a brief report of the IFCT-2105 LURBICLIN study
Source: ESMO Open. 2024 Nov 27;9(12):103968. doi: 10.1016/j.esmoop.2024.103968 (PMC11635664; doi:10.1016/j.esmoop.2024.103968)
Supplement: Supplementary Table S1 [file mmc1.docx]

**SUPPLEMENTAL TABLE**

**Supplemental Table 1:** Safety profile of lurbinectedin

|  |  | |
| --- | --- | --- |
|  | **Grade 3** | **Grade 4** |
|  |  |  |
|  |  |  |
| **Any treatment-related adverse event** | **28 (9.0%)** | **15 (4.8%)** |
|  |  |  |
| **Investigations** | 16 (5.1%) | 13 (4.2%) |
| Platelet count decreased | 13 (4.2%) | 6 (1.9%) |
| Neutrophil count decreased | 5 (1.6%) | 6 (1.9%) |
| Gamma-glutamyl transferase increased | 1 (0.3%) | 3 (1%) |
| Aspartate aminotransferase increase | 2 (0.6%) | 1 (0.3%) |
| Alanine aminotransferase increased | 1 (0.3%) | 1 (0.3%) |
| Blood bilirubin increased | 1 (0.3%) | 0 |
| Lipase increased | 0 | 1 (0.3%) |
| **Blood and lymphatic system disorders** | 7 (2.2%) | 3 (1%) |
| Anemia | 5 (1.6%) | 0 |
| Febrile neutropenia | 2 (0.6%) | 2 (0.6%) |
| Leukocytosis | 0 | 1 (0.3%) |
| **General disorders** | 8 (2.6%) | 0 |
| Fatigue | 8 (2.6%) | 0 |
| **Respiratory** | 2 (0.6%) | 0 |
| Haemoptysis | 1 (0.3%) | 0 |
| Pleural effusion | 1 (0.3%) | 0 |
| Pneumonia | 1 (0.3%) | 0 |
| **Gastrointestinal disorders** | 1 (0.3%) | 0 |
| Nausea | 1 (0.3%) | 0 |
| **Metabolism and nutrition disorders** | 1 (0.3%) | 0 |
| Decreased appetite | 1 (0.3%) | 0 |
| **Musculoskeletal** | 0 | 1 (0.3%) |
| Rhabdomyolysis | 0 | 1 (0.3%) |
|  |  |  |
